# Classifying social anxiety disorder using multivoxel pattern analyses of brain function and structure^☆^

**DOI:** 10.1016/j.bbr.2013.11.003

**Published:** 2014-02-01

**Authors:** Andreas Frick, Malin Gingnell, Andre F. Marquand, Katarina Howner, Håkan Fischer, Marianne Kristiansson, Steven C.R. Williams, Mats Fredrikson, Tomas Furmark

**Affiliations:** a Department of Psychology, Uppsala University, Uppsala, Sweden; b Department of Women's and Children's Health, Uppsala University, Uppsala, Sweden; c Department of Neuroimaging, Centre for Neuroimaging Sciences, Institute of Psychiatry, King's College London, London, UK; d Department of Clinical Neuroscience, Karolinska Institutet, Stockholm, Sweden; e Department of Psychology, Stockholm University, Stockholm, Sweden

**Keywords:** Support vector machine, Classification, Social anxiety disorder, Multivoxel pattern analysis, Biomarker

## Abstract

Functional neuroimaging of social anxiety disorder (SAD) support altered neural activation to threat-provoking stimuli focally in the fear network, while structural differences are distributed over the temporal and frontal cortices as well as limbic structures. Previous neuroimaging studies have investigated the brain at the voxel level using mass-univariate methods which do not enable detection of more complex patterns of activity and structural alterations that may separate SAD from healthy individuals. Support vector machine (SVM) is a supervised machine learning method that capitalizes on brain activation and structural patterns to classify individuals. The aim of this study was to investigate if it is possible to discriminate SAD patients (*n* = 14) from healthy controls (*n* = 12) using SVM based on (1) functional magnetic resonance imaging during fearful face processing and (2) regional gray matter volume. Whole brain and region of interest (fear network) SVM analyses were performed for both modalities. For functional scans, significant classifications were obtained both at whole brain level and when restricting the analysis to the fear network while gray matter SVM analyses correctly classified participants only when using the whole brain search volume. These results support that SAD is characterized by aberrant neural activation to affective stimuli in the fear network, while disorder-related alterations in regional gray matter volume are more diffusely distributed over the whole brain. SVM may thus be useful for identifying imaging biomarkers of SAD.

## Highlights

• Social anxiety disorder (SAD) is a common and disabling psychiatric disorder.
• Support vector machines (SVM) were trained to separate SAD from controls.
• Neural face processing in the fear network separated SAD patients from controls.
• Gray matter volume alterations over the whole brain separated SAD from controls.
• SVM classifiers may be useful for identifying imaging biomarkers of SAD.

## 1 Introduction

Social anxiety disorder (SAD) is a common and disabling condition characterized by anxious feelings of being negatively evaluated or scrutinized in social situations [1], [2]. The disorder is associated with an overly reactive amygdala coupled to an inability to recruit prefrontal inhibitory areas [3], [4], [5], [6]. However, not all studies have found amygdala hyperactivity [7], and functional differences in other brain regions, including the insula and fusiform gyrus, have also been reported, [6], [8]. Recent studies on brain characteristics of SAD have demonstrated structural alterations in a number of temporal and frontal regions as well as in limbic structures, but in comparison to the functional studies, the findings are more mixed with no clear pattern of structural deviation [9], [10], [11], [12], [13]. Thus, to date, no reliable biomarker of SAD has been detected [14].

Analyses of brain imaging data typically use univariate methods to compare conditions or groups. This enables the analysis of differences in neural activity or structure between groups but generally restricts comparison to individual voxels. On the other hand, pattern recognition methods, often referred to as multi-voxel pattern analysis (MVPA) in the neuroimaging context, are inherently multivariate and utilize information distributed across multiple voxels [15]. MVPA is sensitive to spatially distributed effects and can be used to predict both categorical data, separating patients from healthy controls, and continuous outcomes, predicting severity of symptoms or performance at the individual level [16].

One pattern recognition algorithm commonly employed in neuroimaging is the support vector machine (SVM), where a classifier is trained on a subset of the data and then used for a dichotomous prediction of unseen data (referred to as “test” data) into one of two categories (e.g. patients or healthy controls). In an overview of psychiatric and neurologic disorders, SVM was found to successfully separate patients with several disorders such as dementia, mild cognitive impairment, depression and schizophrenia from healthy controls [17], [18]. Both structural [17] and functional [18] imaging have been used to predict psychiatric disorder status with relatively good accuracy (58–86%). Two recent papers have used SVM to separate SAD patients from controls. Liu and colleagues correctly discriminated SAD patients from controls based on resting state functional connectivity [19] while Pantazatos et al., showed that functional connectivity during face processing separated SAD patients from controls, as well as SAD from panic disorder patients [20].

In this study, we used functional and structural magnetic resonance imaging (fMRI and sMRI) in conjunction with SVM to discriminate SAD from healthy controls (HC) based on patterns of (1) neural responses to fearful faces evaluated using blood oxygenation level-dependent (BOLD) fMRI and (2) regional gray matter volume evaluated with sMRI. Analyses were performed with information from the whole brain, as well as separately from fear-relevant regions of interest.

## 2 Methods

### 2.1 Participants

Fourteen male, right-handed patients who met the DSM-IV [1] criteria for SAD (mean ± SD age 32.4 ± 8.8 years) were included together with twelve HC male right-handed subjects (28.0 ± 8.2 years). The mean age did not differ significantly between the two groups (*t*(24) = 1.28, *p* = 0.21). Psychiatric diagnoses were assessed using the Structured Clinical Interview for DSM-IV (SCID-I) [21]. All patients fulfilled the DSM-IV criteria for SAD as primary diagnosis. Two patients had mild or sub-threshold obsessive compulsive disorder (OCD), and one had comorbid snake phobia. The SAD patient diagnosed with comorbid OCD was on a steady dose of venlafaxine. Another of the SAD patients was on varenicline tartrate, and one of the controls was on levothyroxine. None of the HC participants fulfilled criteria for any psychiatric diagnosis, nor did they have a history of psychiatric disorders. For a detailed description of the participants and recruitment procedure, see Frick et al. [9].

### 2.2 Ethic statement

The study was approved by the Regional Ethical Committee at the Karolinska Institutet, Stockholm and conducted in accordance with the Helsinki Declaration. All participants provided written informed consent prior to commencement of the study.

### 2.3 Image acquisition

Structural and functional scans were acquired on a Siemens Avanto 1.5 T whole body MR-scanner equipped with a 12-channel matrix head coil. Participants’ heads were fixated during image acquisition using a vacuum pillow. A 3D magnetization-prepared rapid acquisition gradient echo (MPRAGE) sequence was used to collect structural scans, 176 slices, repetition time (TR) 2300 ms, inversion time (TI) 1100 ms, echo time (TE) 3.93 ms, slice thickness 1 mm, field of view (FOV) 256 mm × 256 mm, matrix 256 × 256. A T2*-weighted gradient echo planar imaging (EPI) sequence was used to acquire functional scans, 30 interleaved coronal slices, 114 volumes, TR 3000 ms, TE 50 ms, slice thickness 5 mm, gap between slices 0.5 mm, FOV 220 mm, matrix 64 × 64, inplane voxel dimension 3.4 mm × 3.4 mm.

### 2.4 Stimuli

During fMRI, participants underwent a standard emotional face paradigm consisting of alternating 36 s long blocks of fearful and neutral faces, including 15 faces presented for 2 s each followed by a fixation cross for 400 ms, interspersed with 18 s long resting blocks showing a white fixation cross on a black background. Participants viewed grayscale photographs of faces from the Ekman and Friesen face collection [22] and were asked to identify the sex of the face. Reaction time and accuracy data were analyzed using between-group *t*-tests with the alpha level set to *p* < .05. Demographic and behavioral data were analyzed with R 2.14.1 (R Foundation for Statistical Computing, Vienna, Austria). Detailed results have been reported in Frick et al. [23]. No significant group differences were found in accuracy or reaction time (all *p* values > .3). The total duration of the paradigm was 5 min and 42 s.

### 2.5 Image preprocessing

Structural and functional MR image preprocessing were carried out using Statistical Parametric Mapping 8 (SPM8; www.fil.ion.ucl.ac.uk/spm) implemented in MATLAB R2012a (The Mathworks Inc., Natick, MA, USA). The first three volumes for each participant were discarded to allow for T1 equilibration effects. Standard image preprocessing steps were performed in the following order: (1) slice timing correction to middle slice, (2) motion correction by realignment of functional volumes to mean volume, (3) coregistration of functional and structural scans, (4) normalization of functional scans to Montreal Neurological Institute (MNI) standard space and reslicing to 3 mm isotropic voxels, and (5) smoothing of functional scans with an 8 mm 3D Gaussian kernel (full width, half maximum).

The BOLD signal was modeled with the general linear model at each voxel using a canonical hemodynamic response function and a 128 s high-pass filter. Fearful and neutral face-blocks were included as regressors in the model together with six realignment parameters from the motion correction step. Brain reactivity to (1) fearful faces over baseline (fixation cross) and (2) fearful over neutral faces were modeled for each participant and used in subsequent SVM analysis.

Regional gray matter volume was calculated from structural T1 weighted images by the following procedure: (1) each participant's T1 weighted image was segmented to gray matter, white matter, and cerebrospinal fluid by use of the new segment routine in SPM8; (2) all participants’ gray and white matter images were used to create a study specific template by use of diffeomorphic anatomical registration through exponentiated lie algebra (DARTEL) [24]; (3) the gray matter images were warped to the study specific template, and in the same step resliced to isotropic 1.5 mm voxels; (4) the warped gray matter images were scaled with the Jacobian determinants; and (5) smoothed with an 8 mm 3D Gaussian kernel. The scaled, smoothed images represent regional gray matter volume, and were used in subsequent SVM analyses.

### 2.6 SVM analyses

SVM analyses were carried out in the Pattern Recognition for Neuroimaging Toolbox (PRoNTo) [16]. In the between-group analyses, SAD patients and HC were entered as two classes in separate SVM analyses with the following images as inputs: (1) contrast images for fearful faces over baseline, (2) contrast images for fearful over neutral faces, and (3) scaled, smoothed gray matter images. Both whole brain and region of interest (ROI) analyses were performed. ROIs included (1) the fear network [5] implicated in SAD pathology i.e. the amygdala, anterior cingulate cortex (ACC), hippocampus, and insula, and (2) the parietal lobe as a non-fear control ROI. In each of the analyses, data were mean centered and leave one subject out cross-validation was performed, making the test set independent from the training set, and the SVM soft margin parameter C was fixed to its default value (one) [16]. Analyses were restricted to voxels where all subjects had non-zero values. Statistical significance of the classifications was tested using permutation testing with 1000 permutations with random assignment of group class to input image. The resulting null-hypothesis distribution was used to calculate the *p*-value of the accuracies, i.e. the proportion of permutations that yielded a greater accuracy than the accuracy found for the classification models.

## 3 Results

### 3.1 BOLD signal changes

**Fig. 1:**
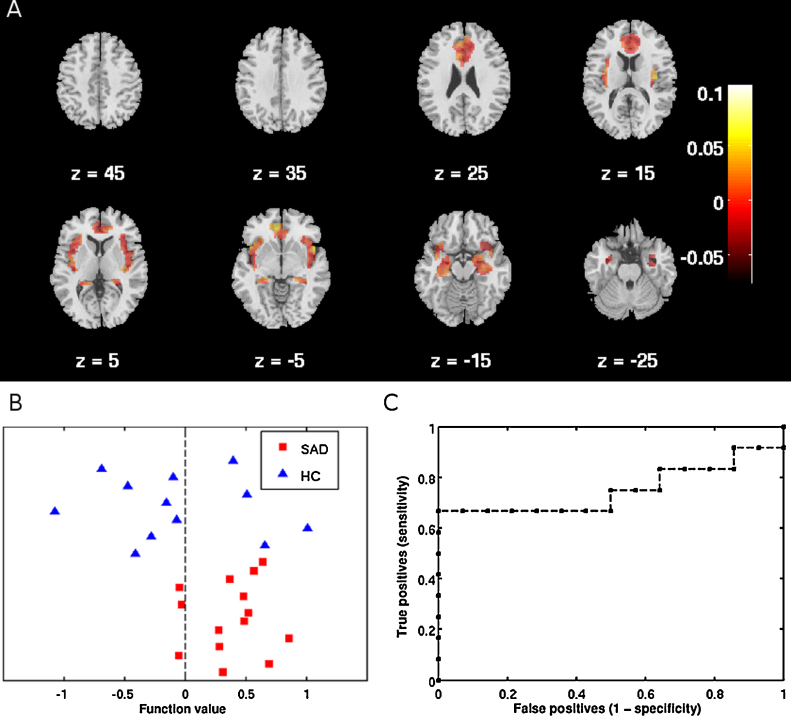
Support vector machine classification of patients with social anxiety disorder (SAD) and healthy controls (HC) based on functional changes in blood oxygenation level-dependent signal to fearful faces in the fear network including the amygdala, anterior cingulate cortex, hippocampus and insula cortex. (A) Weight map indicating relative weights ascribed to voxels at representative transverse slice levels in mm (MNI) as indicated by Z. Colorbar indicates weights. (B) Classification of SAD and HC participants. Positive function values for SAD patients indicate true positives. Negative function values for HC participants indicate true negatives. (C) Receiver operating characteristic (ROC) curve showing the trade-off between sensitivity and specificity, including area under the curve (AUC = 0.75). (For interpretation of the references to color in figure legend, the reader is referred to the web version of the article.)

SVM analyses with the fMRI contrast images of fearful faces over baseline resulted in a significant balanced accuracy of 72.6% for classification of SAD and HC when restricting the analysis to the fear network (see Table 1 and Fig. 1). Using information from the whole brain yielded a significant balanced accuracy of 68.5%. SVM analysis based on reactivity in the parietal lobe did not yield significant classifications.

**Table 1:** Classification accuracies in percent for social anxiety disorder (SAD), healthy controls (HC), and balanced accuracy of support vector machine analyses separation of SAD from HC based on functional imaging of BOLD response to fearful faces.

	SAD	*P* (SAD)^a^	HC	*P* (HC)^a^	Balanced accuracy	*P* (Balanced)^a^	AUC^b^
Whole brain	**78.6**	**0.012**	58.3	0.075	**68.5**	**0.034**	0.70
Fear network (amygdala, ACC^c^, hippocampus, insula)	**78.6**	**0.007**	**66.7**	**0.034**	**72.6**	**0.017**	0.75
Parietal lobe	50.0	0.662	41.7	0.605	45.8	0.548	0.45

Significant accuracies in bold print, as determined by permutation testing.

a *P*-values are calculated from permutation testing with 1000 permutations.

b Area under the receiver operating characteristic curve.

c Anterior cingulate cortex.

We also performed the analyses with the contrast images fearful over neutral faces, resulting in non-significant classification accuracies for the fear network (balanced accuracy: 45.8%, *p* = 0.552; SAD: 50.0%, *p* = 0.649; HC: 41.7%, *p* = 0.615), the parietal lobe (balanced accuracy: 53.0%, *p* = 0.333; SAD: 64.3%, *p* = 0.299; HC: 41.7%, *p* = 0.571), and the whole brain (balanced accuracy: 61.3%, *p* = 0.124; SAD: 64.3%, *p* = 0.322; HC: 58.3%, *p* = 0.168).

### 3.2 Regional gray matter volume

**Fig. 2:**
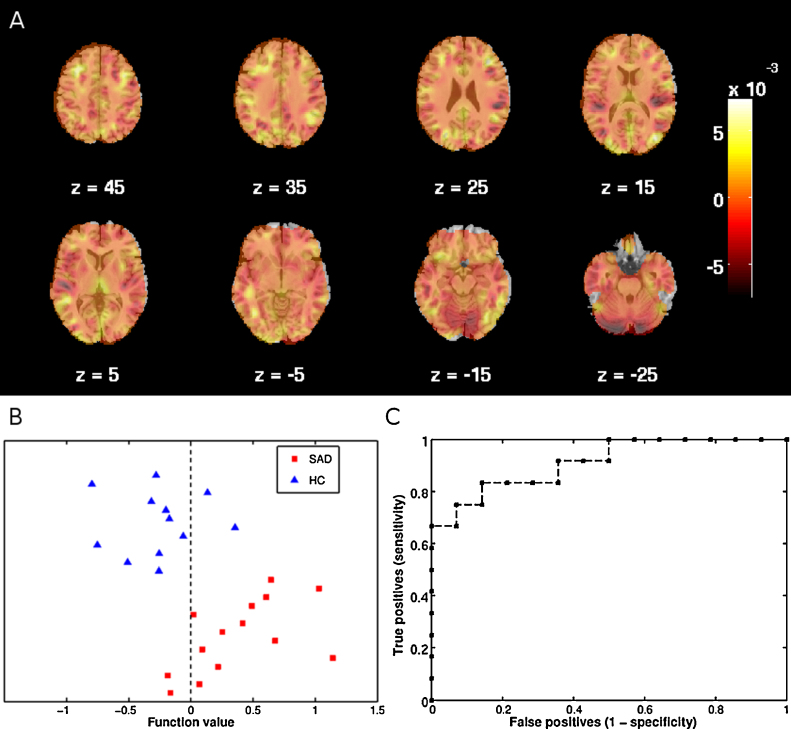
Support vector machine classification of patients with social anxiety disorder (SAD) and healthy controls (HC) based on regional gray matter volume. (A) Weight map indicating relative weights ascribed to voxels at representative transverse slice levels in mm (MNI) as indicated by Z. Colorbar indicates weights. (B) Classification of SAD and HC participants. Positive function values for SAD patients indicate true positives. Negative function values for HC participants indicate true negatives. (C) Receiver operating characteristic (ROC) curve showing the trade-off between sensitivity and specificity, including area under the curve (AUC = 0.91). (For interpretation of the references to color in figure legend, the reader is referred to the web version of the article.)

By entering regional gray matter volume images, a balanced accuracy of 84.5% correct classification was achieved with information from the whole brain (see Table 2 and Fig. 2). Restricting the analyses to the fear network and the parietal lobe did not produce significant classification accuracies in any case.

**Table 2:** Classification accuracies in percent for social anxiety disorder (SAD), healthy controls (HC), and balanced accuracy of support vector machine analyses separation of SAD from HC based on regional gray matter volume imaging.

	SAD	*P* (SAD)^a^	HC	*P* (HC)^a^	Balanced accuracy	*P* (Balanced)^a^	AUC^b^
Whole brain	**85.7**	**0.002**	**83.3**	**0.001**	**84.5**	**0.001**	**0.91**
Fear network (amygdala, ACC^c^, hippocampus, insula)	50.0	0.466	50.0	0.238	50.0	0.397	0.39
Parietal lobe	64.3	0.268	50.0	0.394	57.1	0.232	0.51

Significant accuracies in bold print, as determined by permutation testing.

a *P*-values are calculated from permutation testing with 1000 permutations.

b Area under the receiver operating characteristic curve.

c Anterior cingulate cortex.

## 4 Discussion

SVM analyses using either functional or structural imaging both yielded significant classifications with accuracies comparable to studies on other psychiatric disorders [18] indicating that SVM is a feasible method to discriminate between SAD and HC. Neural face processing in the fear network alone correctly discriminated between SAD and HC, while the structural analyses yielded significant classification accuracy only when utilizing information from the whole brain. The results suggest that SAD may be characterized by altered neural activity in the fear network during fearful face processing and more distributed structural alterations over the whole brain.

Concerns have been raised that social anxiety is over-pathologized and lacks reliable biomarkers [14]. However, our results, where brain reactivity and gray matter volume could be used to distinguish between patients and controls support that SAD is a brain linked disorder, and that weights from the SVM classifiers may serve as biomarkers [17]. This is in line with two recent reports of discrimination of SAD from controls using SVM based on functional connectivity during rest or face processing [19], [20]. In earlier work, univariate methods were used to predict therapy outcome in SAD [25]. In light of the present results, it is possible that SVM could add value to individual treatment response predictions. In depression, a recent study successfully used pattern recognition on functional brain scans collected at pre-treatment to predict subsequent treatment responders [26]. Thus, a logical next step would be to evaluate if pattern analyses could be used to predict treatment outcome in patients with SAD.

The functional alterations reflected activity during implicit processing of fearful emotional faces. This task was chosen as previous studies of SAD have demonstrated differential activations in SAD and HC during evaluation of emotional faces [6], [27]. Admittedly, these prior findings were based on processing of fearful in contrast to neutral faces. In this study, discrimination of SAD patients from HC was successful only when using fearful faces in contrast to fixation baseline, in line with earlier classification studies in psychiatric disorders [26], [28]. Even though activity in temporal and prefrontal areas occasionally may separate SAD from HC [7], [23], the most consistent differences in reactivity between patients and controls have been noted in the fear network [5], [6]. In line with this, reactivity to fearful facial stimuli in the fear network but not in the parietal lobe discriminated between SAD and HC. Likewise, earlier structural studies using voxel-wise analyses have found structural alterations in SAD in a number of regions in the temporal and frontal cortices as well as in limbic structures, but findings are inconclusive with regards to anatomical localization [9], [12], [13]. In the present study, SVM yielded high classification accuracy from structural information in the whole brain, but not when restricting classifications to ROIs, possibly indicating that structural changes in SAD may be diffusely distributed rather than localized to specific regions.

A limitation of the current study is the relatively small sample size. Interpretation of the sensitivity and specificity for the classifications should thus be done with caution. Furthermore, since only males were included, some of the sex-specific variance was reduced, possibly increasing prediction accuracies. Also, while facial stimuli are frequently used in SAD fMRI research, other tasks might result in different classification accuracies from BOLD signal changes. Indeed our functional SVM results were task-dependent since we only achieved significant classification accuracies when using the contrast fearful faces over baseline, but not when using the contrast fearful over neutral faces.

## 5 Conclusions

In conclusion, the current results support the notion that SAD is a brain linked disorder, neurally characterized by aberrant emotional face processing in the fear network but with more diffuse deviations in regional gray matter volume over the whole brain. Thus, support vector machines based both on functional and structural brain imaging data were able to discriminate between SAD patients and controls, indicating that this method can be used to identify diagnostic biomarkers.
